# Concurrent Afatinib and Whole-Brain Radiotherapy in Exon 19-del-EGFR Mutant Lung Adenocarcinoma: A Case Report and Mini Review of the Literature

**DOI:** 10.3389/fonc.2017.00088

**Published:** 2017-05-10

**Authors:** Chukwuka Eze, Nina-Sophie Hegemann, Olarn Roengvoraphoj, Maurice Dantes, Farkhad Manapov

**Affiliations:** ^1^Department of Radiation Oncology, LMU Munich, Munich, Germany

**Keywords:** afatinib, brain and leptomeningeal metastases, non-small cell lung cancer, tyrosine-kinase inhibitor, whole-brain radiotherapy

## Abstract

Leptomeningeal metastases (LM) are found in approximately 3.8% of non-small cell lung cancer cases with an increased incidence in adenocarcinoma, and approximately one-third of patients will present with concomitant brain metastases. We report the case of a 50-year-old male patient with stage IV exon 19-del-EGFR mutant lung adenocarcinoma who progressed on second-generation TKI therapy with manifestation of symptomatic simultaneous diffuse brain and LM. Whole-brain radiotherapy with concurrent afatinib resulted in an almost complete regression of neurological symptoms as well as good, durable radiological response. Furthermore, treatment was well tolerated with no relevant adverse effects.

## Introduction

Leptomeningeal metastases (LM) represent the infiltration of the leptomeninges by malignant cells (1) and are a readily increasing complication in cancer patients. LM are found in approximately 5% of patients with malignant tumors (1), and in non-small cell lung cancer (NSCLC), the incidence is approximately 3.8% with an increased incidence in adenocarcinoma, and roughly one-third of patients will present with concomitant brain metastases (BM) (2). The incidence is steadily increasing due to improvement in diagnostic tools such as neuroimaging methods as well as availability of improved treatments, consecutively leading to improved survival. However, general prognosis remains poor in the order of 2–4 months (3–7). Currently, no standard treatment exists (8). Occurrence of LM under prolonged tyrosine-kinase inhibitor (TKI) therapy has been previously described (9).

Afatinib is a second-generation irreversible covalent inhibitor of the epidermal growth factor receptor (EGFR) tyrosine kinases including ErbB-2 (HER2) and ErbB-4. Subgroup analysis from LUX-LUNG 3 and LUX-LUNG 6 studies demonstrated a significant overall survival benefit for afatinib compared to chemotherapy in UICC stage IIIB/IV lung adenocarcinoma patients with 19del-EGFR mutation (10). Consecutively, in a combined *post hoc* analysis of both studies, progression free survival was significantly improved with afatinib vs. with chemotherapy in patients with BM (8.2 vs. 5.4 months; HR, 0.50; *p* = 0.0297) (11). There is a paucity of literature on concurrent radiotherapy and second-generation TKIs. In 2014, Atmaca et al. published a case report of a patient who received concomitant radiotherapy to the mediastinum and primary lung tumor with afatinib. The therapy resulted in partial remission in the irradiated sites without any relevant treatment-associated adverse effects (12). In addition, Li et al. reported in a case series on a 52-year-old male patient with BM from an L858R mutant adenocarcinoma of the lung. Whole-brain radiotherapy (WBRT) was delivered to a total dose of 30.0 Gy in 10 fractions, and afatinib was administered concurrently. Follow-up magnetic resonance imaging (MRI) showed regression of BM (13).

## Case

**Figure 1:**
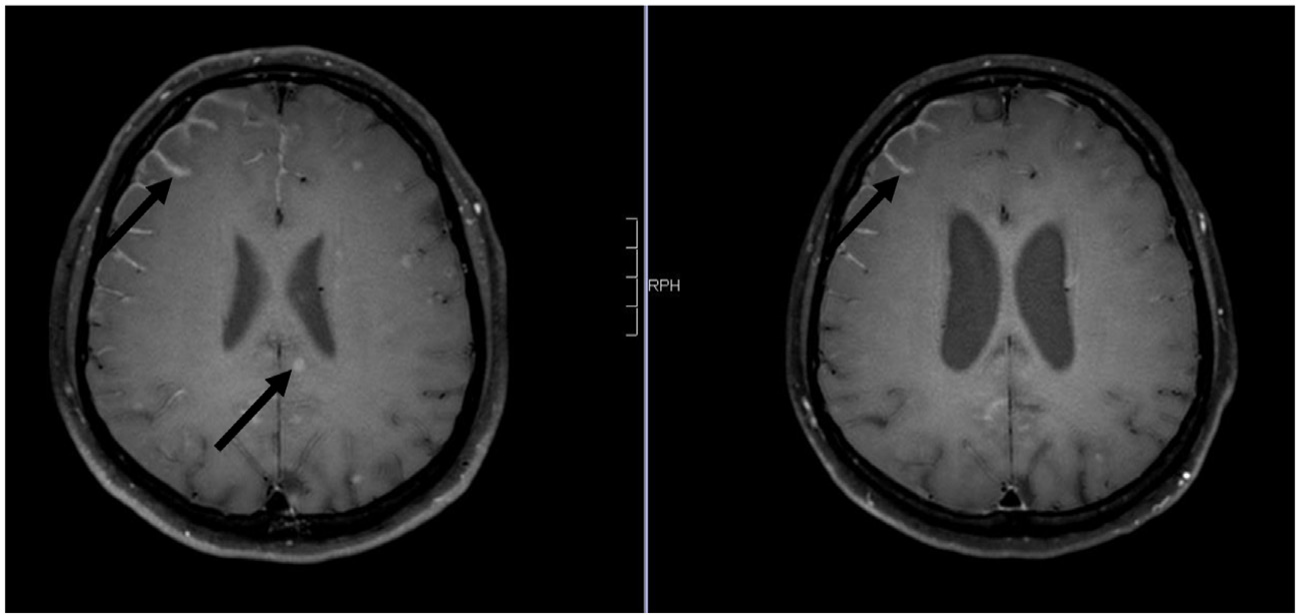
**Axial contrast-enhanced 3D-FAST SPIN ECHO spectral presaturation with inversion recovery [left: pre-whole-brain radiotherapy (WBRT); right: 9-month follow-up]: note decrease in intraparenchymal brain metastases and leptomeningeal enhancement pre- vs. post-WBRT**. In addition, note consecutive enlargement of the lateral ventricles on 9-month follow-up scan.

**Figure 2:**
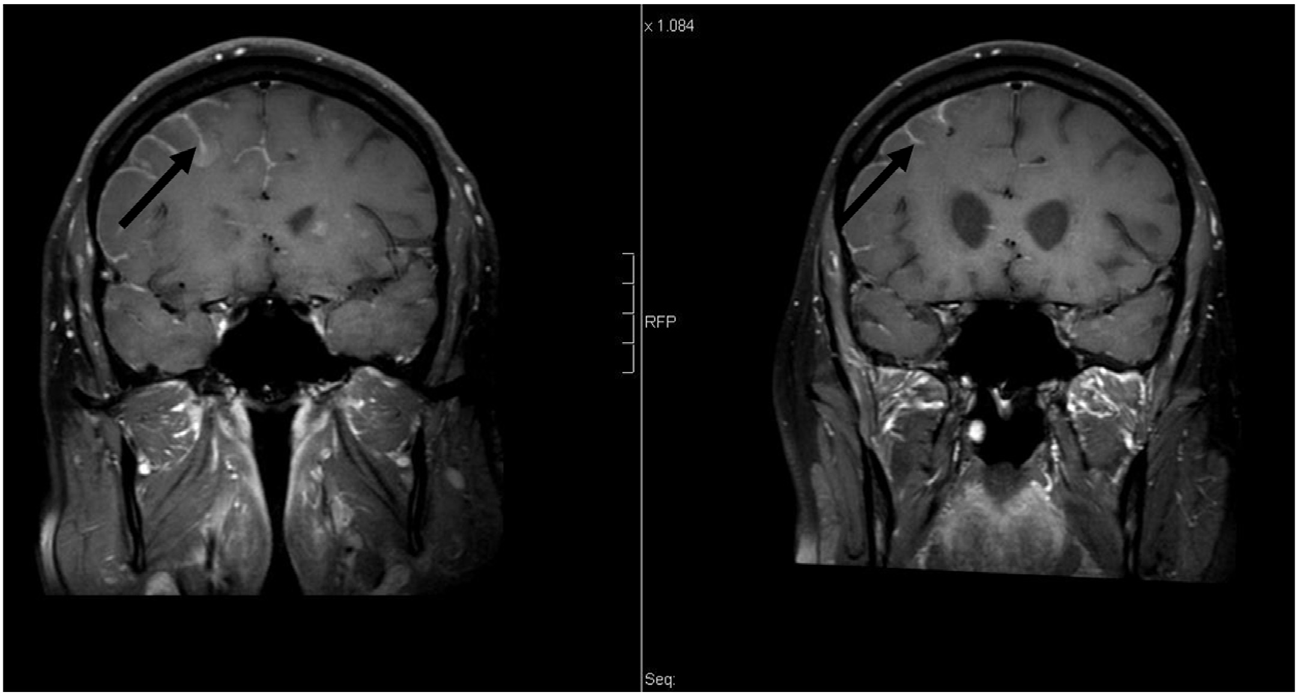
**Coronal contrast-enhanced T1-weighted magnetic resonance imaging [left: pre-whole-brain radiotherapy (WBRT); right: 9-month follow-up]: note decrease in leptomeningeal enhancement pre- vs. post-WBRT**. In addition, note enlargement of the lateral ventricles on 9-month follow-up scan.

**Figure 3:**
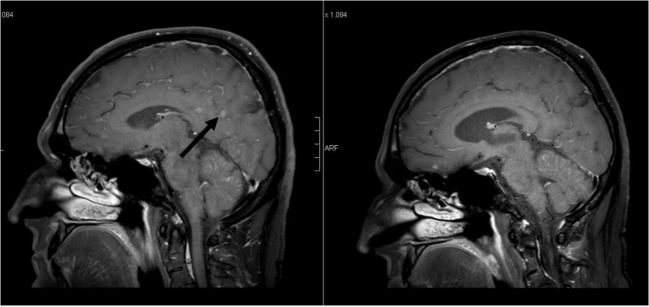
**Sagittal contrast-enhanced 3D-FAST SPIN ECHO spectral presaturation with inversion recovery [left: pre-whole-brain radiotherapy (WBRT); right: 9-month follow-up]: note decrease in intraparenchymal brain metastases enhancement pre- vs. post-WBRT**. In addition, note enlargement of the ventricles on 9-month follow-up scan.

The case of a 50-year-old male never smoker with stage IV Exon 19-del-EGFR mutant, ALK- and ROS-1-negative lung adenocarcinoma diagnosed in April 2015 was presented at the multidisciplinary tumor board. At initial diagnosis, the patient presented with malignant pleural effusion and FDG-avid diffuse bone metastasis on 18F-FDG/PET-CT (positron emission tomography with 2-deoxy-2-[fluorine-18]fluoro-d-glucose integrated with computed tomography). Due to Exon-19-deletion, afatinib 40 mg/day was prescribed. Follow-up imaging showed good extracranial remission. Approximately 12 months later, the patient presented with cephalalgia, seizures, impaired vision, and hypesthesia. Gadolinium-enhanced multiplanar MRI of the brain and spine showed isolated intraparenchymal and leptomeningeal carcinomatosis of the brain; the findings were conclusive for leptomeningeal spread (see Figures 1–3). Analysis of the cerebrospinal fluid (CSF) confirmed the findings. The patient was started on oral dexamethasone. 18F-FDG-PET/CT confirmed no signs of extracranial tumor progression. The patient underwent WBRT with concurrent afatinib due to fear of extracranial tumor progression in the case afatinib was discontinued. WBRT was applied with 6 MV photon beams once daily; fourteen fractions to a total dose of 35.0 Gy and was well tolerated without any severe cutaneous adverse effects. Three-month follow-up MRI and 18F-FDG-PET/CT showed significant regression of intracranial disease as well as stable extracranial disease. Furthermore, the patient noted an almost complete regression of the above-mentioned neurological symptoms. Approximately nine months following WBRT (see Figures 1–3), contrast-enhanced MRI of the brain showed sustained durable response in the absence of any severe neurological side effects. Furthermore, follow-up 18F-FDG/PET-CT showed stable extracranial disease with multiple FDG non-avid sclerotic bone lesions as a sign of treatment response to afatinib. In addition, although there were no signs of tumor progression on the follow-up scans, after discussion at the multidisciplinary tumor board, precautionary further analysis of the CSF extracted at initial diagnosis of cranial metastases for acquired secondary T790M mutation was recommended.

## Discussion

Based on the National Comprehensive Cancer Network guidelines, WBRT is a mainstay in the treatment of BM. However, its role in the treatment of LM is yet to be clearly defined. Morris et al. demonstrated in a retrospective study on LM from NSCLC poor survival. In the study, survival was not improved by WBRT (14). In contrast, Soon et al. showed in a systematic review and meta-analysis that there was some evidence that upfront cranial radiotherapy might improve intracranial disease and survival outcomes compared with TKI alone in patients with EGFR mutant NSCLC (15). A recently published study by Jiang et al. demonstrated no overall survival benefit when first-generation TKIs combined with WBRT vs. TKIs alone were administered (16). Hoffknecht et al. showed that patients pretreated with first-generation TKI and new intracranial progression could benefit from treatment conversion to afatinib (17).

In the present case, simultaneous intraparenchymal brain and LM arose during afatinib therapy despite extracranial tumor control, which can be partially attributed to EGFR mutation heterogeneity (18). There has been no study focusing on the efficacy of WBRT combined with afatinib in LM, but clinical studies on BM have shown that WBRT could increase the permeability of TKI into the CSF (19, 20); hence, in this case, response may also be partially attributed to this phenomenon. In the present case, concomitant afatinib with WBRT was feasible with achievement of rash palliation of symptoms and good, durable radiological response in an Exon19-del EGFR-mutant lung adenocarcinoma patient who progressed during TKI therapy with the manifestation of simultaneous brain and LM. Remarkably, WBRT in combination with afatinib resulted in sustained tumor control going on 9 months following treatment at a stage of disease with dismal prognosis.

## Ethics Statement

This case report was carried out in accordance with the recommendations of the Ethics Committee of the Ludwig Maximilian University of Munich with express written informed consent from the subject, who gave written informed consent in accordance with the Declaration of Helsinki. Treatment was given in palliative intent, hence the exemption from ethics committee approval. This registry does not meet the WHO definition of a clinical trial and is considered exempt from http://clinicaltrials.gov requirements.

## Author Contributions

CE drafted, wrote, and edited the manuscript. N-SH edited and critically revised the manuscript. OR edited the manuscript and figures. MD edited the manuscript. FM edited and critically revised the manuscript for valuable intellectual content.

## Conflict of Interest Statement

The authors declare that the research was conducted in the absence of any commercial or financial relationships that could be construed as a potential conflict of interest. The reviewer, JB, and handling editor declared their shared affiliation, and the handling editor states that the process nevertheless met the standards of a fair and objective review.
